# Mitochondrial genome annotation with MFannot: a critical analysis of gene identification and gene model prediction

**DOI:** 10.3389/fpls.2023.1222186

**Published:** 2023-07-04

**Authors:** B. Franz Lang, Natacha Beck, Samuel Prince, Matt Sarrasin, Pierre Rioux, Gertraud Burger

**Affiliations:** Robert Cedergren Center for Bioinformatics and Genomics, Département de Biochimie, Université de Montréal, Montréal, QC, Canada

**Keywords:** mitochondrial DNA, genome annotation, introns, profile HMMs, ERPIN, covariance models, RNA structure

## Abstract

Compared to nuclear genomes, mitochondrial genomes (mitogenomes) are small and usually code for only a few dozen genes. Still, identifying genes and their structure can be challenging and time-consuming. Even automated tools for mitochondrial genome annotation often require manual analysis and curation by skilled experts. The most difficult steps are (i) the structural modelling of intron-containing genes; (ii) the identification and delineation of Group I and II introns; and (iii) the identification of moderately conserved, non-coding RNA (ncRNA) genes specifying 5S rRNAs, tmRNAs and RNase P RNAs. Additional challenges arise through genetic code evolution which can redefine the translational identity of both start and stop codons, thus obscuring protein-coding genes. Further, RNA editing can render gene identification difficult, if not impossible, without additional RNA sequence data. Current automated mito- and plastid-genome annotators are limited as they are typically tailored to specific eukaryotic groups. The MFannot annotator we developed is unique in its applicability to a broad taxonomic scope, its accuracy in gene model inference, and its capabilities in intron identification and classification. The pipeline leverages curated profile Hidden Markov Models (HMMs), covariance (CMs) and ERPIN models to better capture evolutionarily conserved signatures in the primary sequence (HMMs and CMs) as well as secondary structure (CMs and ERPIN). Here we formally describe MFannot, which has been available as a web-accessible service (https://megasun.bch.umontreal.ca/apps/mfannot/) to the research community for nearly 16 years. Further, we report its performance on particularly intron-rich mitogenomes and describe ongoing and future developments.

## 1 Introduction

Mitochondria and plastids are semi-autonomous organelles of eukaryotic cells endowed with their own genome and molecular machineries for replication, transcription, and translation. While mitochondria originated from bacterial endosymbionts related to extant α-Proteobacteria, plastids share ancestry with Cyanobacteria. Across eukaryotes, the genomes of mitochondria (mtDNA) and plastids (ptDNA) vary considerably in size, architecture, and coding capacity. MtDNA encodes 5 to 100 genes, which play a role in oxidative phosphorylation, protein synthesis, protein transport and maturation, RNA processing, and in some rare instances, transcription (Lavrov and Lang, 2013). PtDNAs encode the same types of genes plus those involved in photosynthesis, summing up to as many as 250 genes (Muñoz-Gómez et al., 2017; de Vries and Archibald, 2018).

Sequencing and complete assembly of eukaryotic organelle genomes has become routine and affordable. Yet, despite the relatively small coding capacity of organelle genomes compared to nuclear genomes, identifying genes and subsequently inferring their internal structure (e.g., exon-intron boundaries, herein referred to as ‘gene modelling’) can be challenging and time-consuming. Indeed, whereas organelle genome annotation typically involves automated gene prediction tools, manual analysis and curation by skilled experts are usually necessary to produce accurate results. In the case of mtDNA, the challenges stem from numerous Group I and Group II introns, twintrons (Hafez et al., 2013b), difficult-to-recognize mini-exons, marginally conserved genes, such as *rps3*, *rnpB*, *ssrA* (Bullerwell et al., 2000; Seif et al., 2003; Hafez et al., 2013a; Donath et al., 2019), and structurally reduced rRNAs and tRNAs (Okimoto et al., 1994). Furthermore, intron identification and classification is often only possible using elaborate and manually-refined computational models (Prince et al., 2022).

Several tools have been developed to annotate organelle genomes, including DOGMA (Wyman et al., 2004), MOSAS (Sheffield et al., 2010), MITOS2 (Donath et al., 2019), Mitofy (Alverson et al., 2010), AGORA (Jung et al., 2018), GeSeq (Tillich et al., 2017), and MFannot (Beck and Lang, 2010). These tools have varying strengths and limitations and are specialized for different groups of organisms. DOGMA and MOSAS were the first to be developed for bilaterian animals. Yet, they produced incomplete gene models requiring substantial expert intervention for completion, and often failed to detect genes outside animals. The more recent tool MITOS2, also tailored to animals, has significantly improved prediction capabilities due to probabilistic inference methods (profile HMMs, CMs) for recognizing protein and ncRNA genes (Bernt et al., 2013; Donath et al., 2019). However, MITOS2 cannot model introns. Although rare in metazoans, introns are present in e.g., corals and sponges (Lang and Burger, 2012; Bernt et al., 2013; Lavrov and Pett, 2016; Donath et al., 2019; Prince et al., 2022). The tools Mitofy, AGORA, and GeSeq were initially optimized for plant organelle genomes which remains their principal strength.

Unfortunately, expert curation of results generated by the above-mentioned tools is not always performed. Consequently, a number of published mitogenomes, even records in the widely used NCBI RefSeq repository (Pruitt et al., 2003), contain latent errors and deficiencies. Moreover, using such data for novel mitogenome annotations inherently propagates errors and deficiencies, particularly in the case of computational methods that use pairwise similarity searches. The obvious drawbacks of this situation are that researchers who download sequences for various comparative analyses and phylogenomics must curate datasets thoroughly.

A critical component of genome annotators is the algorithms employed for gene-model inference. All the tools mentioned above, except MITOS2 and MFannot, heavily use BLAST-like algorithms to search for sequence similarity with known genes, an approach that often has insufficient sensitivity and precision. A more suited approach involves profile HMMs (Eddy, 1995), i.e., Hmmsearch for proteins (Eddy, 2009; Eddy, 2011), Cmsearch for ncRNA genes and introns [Infernal, (Nawrocki and Eddy, 2013)] or as an alternative to Cmsearch, ERPIN (Lambert et al., 2004; Prince et al., 2022). Among current organelle annotators, only MITOS2 and MFannot use profile HMMs, Infernal or ERPIN (GeSeq applies HMMs only for prediction refinements).

MFannot, developed in our laboratory, is a comprehensive mitochondrial genome annotation pipeline available as a stand-alone software and a web service. It is optimized for annotating mitogenomes of eukaryotes other than bilaterian animals, but is also capable of annotating plastid genomes (although less accurately than GeSeq). For modelling of intron-containing protein-coding genes, MFannot employs Exonerate (Slater and Birney, 2005) and Hmmsearch (Eddy, 2009). In contrast, the tools used for identifying ncRNAs (including RNase P RNAs, 5S rRNAs and tmRNAs) are Infernal and ERPIN (Lang et al., 2007; Hafez et al., 2013a; Valach et al., 2014). MFannot is unique compared to other annotators for employing probabilistic intron prediction, and its capability to detect mini-exons that are difficult to recognize with other tools.

## 2 Results and discussion

### 2.1 Two conceptually distinct approaches to organelle genome annotation

From an algorithmic point of view, organelle genome annotators come in two different flavours, one of which is a next-neighbour-guided annotation (e.g., DOGMA, MOSAS, Mitofy, AGORA, GeSeq), i.e., the identification of genes and genetic elements through comparison with and transfer from well-annotated genomes of very closely related species. For this, BLAST-type similarity-search algorithms (e.g., BLAST and Diamond (Altschul et al., 1990; Buchfink et al., 2015)) are well suited. The advantage of next-neighbour-guided annotation is its very fast computational speed. However, it critically relies on a large and taxonomically broad collection of essentially error-free and complete genome annotations. Hence, the drawback is that this approach is prone to perpetuating occasional annotation errors and gene omissions. The procedure is also less effective for species in which well-annotated mitogenomes from close neighbours are currently unavailable. Therefore, high-quality expert curation of a large number and phylogenetically broad collection of model mitogenomes is a prerequisite this approach.

The alternative to next-neighbour guided annotation is the *ab-initio* inference of gene models using probabilistic methods (widely employed by MITOS2 and MFannot). For this, sensitive sequence search algorithms (profile HMMs, ERPIN, CMs), based on accurate and evolutionary broad multiple sequence alignments, are employed to identify and model even marginally conserved sequences without requiring annotated genomes of close relatives. This approach comes with longer computation times, notably 1-2 h for a small bilaterian mitogenome by MITOS2 (Bernt et al., 2013), but on the other hand, a reasonable 2-10 min for mitogenomes of ~20-200 kbp with the current version of MFannot. (Note that future versions will likely require more execution time, particularly for inferring complete gene structures of rRNA-encoding genes). Significant advantages of *ab-initio* approaches are high-quality genome annotations for species without close-neighbour information and a moderate requirement for expert curation.

### 2.2 RNA mapping evidence is of limited value in mitochondrial gene-model prediction

**Figure 1:**
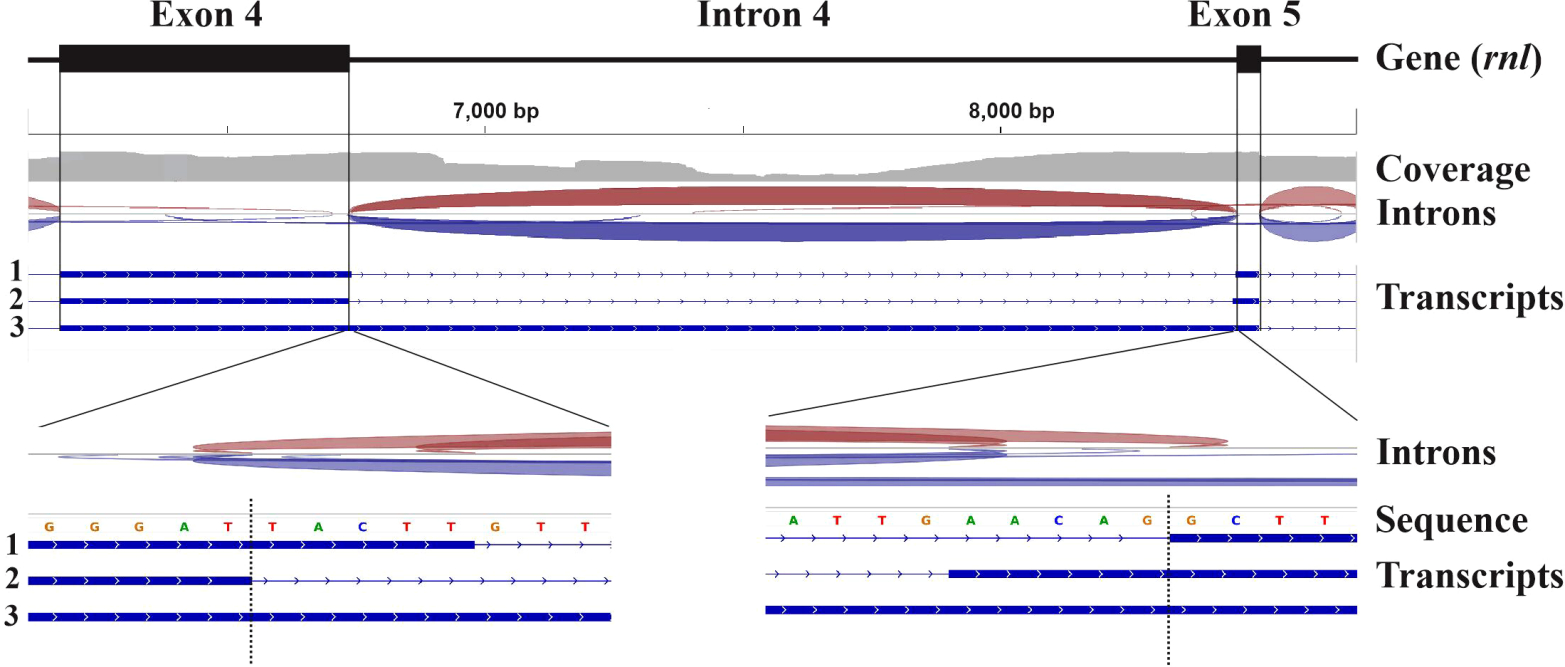
Mapping of RNA-seq data to the mitochondrial genome of *Coccidioides posadasii*. The figure depicts a 2,579 nt window over the coordinates of exons 4 (6172-6736) and 5 (8458-8504) of the mitochondrial gene encoding the large subunit rRNA (*rnl*) of *C. posadasii* (see (de Melo Teixeira et al., 2021) for details on genome sequencing) as determined by rRNA sequence conservation and structural modelling of exons 4 and 5 plus adjacent introns. The read-mapping coverage distribution (grey) is shown below the annotated exons. Red and blue arches indicate forward and reverse split reads, respectively. The bottom part of the upper panel depicts the splice junctions inferred from the RNA-Seq read to genome mapping. The coverage profile reveals a substantial number of reads that map within intron 4 due to stable transcripts encoding intron ORFs and an elevated level of un-spliced RNA precursors, which is common in mitochondria. The two distinct splice junctions inferred at the 5’ and 3’ end of intron 4 are supported in roughly equal proportions by mapped reads. The bottom track depicts exons inferred from three distinct transcripts (marked 1, 2 and 3) that were reconstructed from the mapped reads using StringTie. The two lower panels show read mapping in higher resolution. The vertical dotted line indicates the predicted precise splice junction.

In contrast to nuclear genome annotation approaches, none of the (above-mentioned) organelle genome annotators use RNA data. Furthermore, RNA data are not reported in most organelle genome publications. The reason lies in the particularities of organelle transcript processing and intron splicing. Only a few species produce a high proportion of fully mature mitochondrial transcripts [e.g., the fungus *Schizosaccharomyces pombe* (Schafer et al., 2005; Shang et al., 2018)], to be used to infer intron and gene boundaries from RNA-seq read coverage. In most other species, the transcript landscape is highly complex, particularly for intron-containing genes (e.g., *Saccharomyces cerevisiae* and most other fungal mtDNAs rich in introns). The complexity of the observed transcript population is due to the stability of intron RNAs that encode proteins [e.g., (Anziano et al., 1982; Hanson et al., 1982; Turk et al., 2013)] or form thermodynamically stable RNA structures, as well as slow and partial intron splicing (**Figure 1**). Together, this leads to highly variable coverage of RNA-seq reads, often spanning exon-intron boundaries. As a result, mapping RNA-seq reads or splice-aligning assembled transcripts to the genome sequence often generates conflicting information that interferes with or misleads gene modelling. In the example shown in **Figure 1** [data from (de Melo Teixeira et al., 2021)], expert inference of the gene structure was only possible by sequence comparison with intron-less gene homologs in related species.

### 2.3 The MFannot annotation procedure

MFannot is written in Perl and was designed to annotate protein-coding and ncRNA genes in mitochondrial and chloroplast genomes. It uses the RNA/intron detection tools described below and is particularly helpful when organelle genomes contain numerous introns. Intron-exon boundaries of protein-coding genes are identified by sequence conservation of exons together with profiles of Group I and II intron-splice sites that, in most instances, can be precisely inferred without transcript data. The output of MFannot lists gene coordinates either in a format that can be directly loaded into NCBI sequence submission tools or in ‘masterfile’ format (a computer-parsable and simultaneously human-readable format developed in-house, with annotations embedded into the sequence as comment lines).

**Figure 2:**
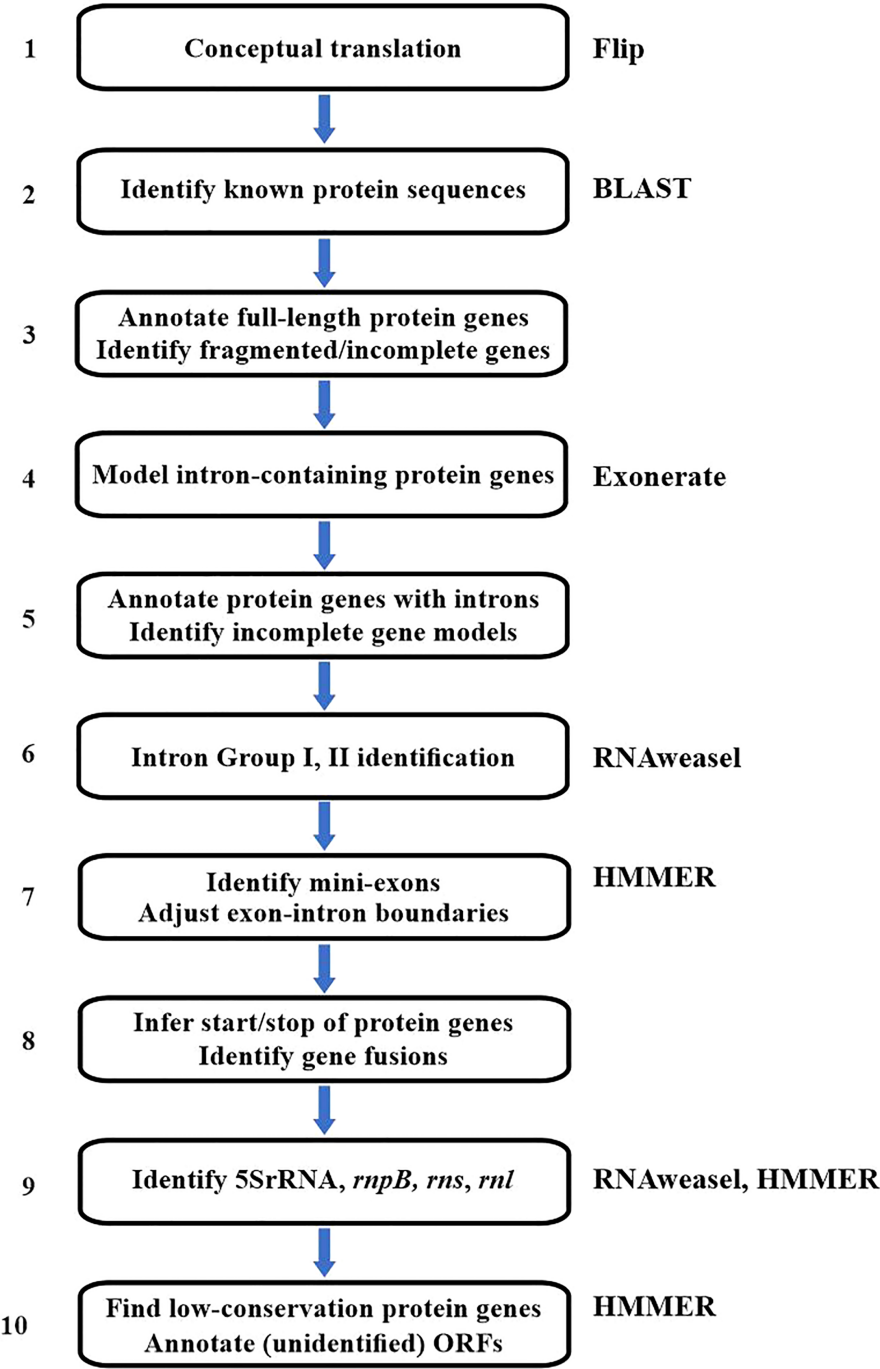
Flowchart of the MFannot annotation procedure. The figure summarizes the analytical steps and the external tools employed (indicated to the right). The external tools are Flip (conceptual translation), BLAST (fast sequence similarity search), Exonerate (annotation of genes with exons/intron structure), HMMER (high sensitivity sequence matching), and RNAweasel (introns Group I and II, ncRNAs). When no specific tool is mentioned, the corresponding step is executed by MFannot-specific code. The RNAweasel tool (step 6 and 9) uses ERPIN (and more recently, Infernal) as a search engine. ERPIN’s search algorithm is based on RNA secondary structure profiles computed from RNA sequence alignments plus user-defined secondary-structure information as an input. Much of ERPIN’s efficiency stems from the definition of precisely delimited structural elements that can be searched individually or in combination using a particular search order (‘search strategy’). In general, ERPIN (Gautheret and Lambert, 2001) is the second-most sensitive search algorithm for structured RNAs [following the covariance-based Infernal program (Nawrocki and Eddy, 2013)]. Still, it distinguishes more reliably certain mitochondrial Group I and II introns.

The current annotation procedure (**Figure 2**) starts with the conceptual translation of the mitogenome (step 1) into Open Reading Frames (ORFs) ≥40 amino acids long, using an in-house tool called Flip (Brossard et al., 1996). For this, the user supplies the genetic code. The genetic code in mitochondria varies substantially [e.g., (Su et al., 2011; Ling et al., 2014)] and should be verified case-by-case, and may require the analysis of potential codon deviations with a dedicated tool (Noutahi et al., 2017).

In step 2, all predicted ORFs are searched with BLAST against a broad collection of known mitochondrial and plastid proteins, to identify known organellar genes as well as typical intron-encoded ORFs (nucleases, intron maturases, reverse transcriptases) and genes (in particular those encoding RNA and DNA polymerases) derived from the insertion of mitochondrial plasmids into the mitogenome.

In step 3, MFannot uses full-length BLAST matches directly for annotating the respective genes. In addition, MFannot will identify and flag potential frameshifts indicative of either a pseudo-gene or sequencing errors. Partial matches indicate intron insertions, incomplete genes, or trans-splicing (i.e., exons encoded on different DNA strands or at positions that are too distant to account for intron insertions) and are dealt with in subsequent steps.

Notably, the information on the location of *bona fide* intron-containing genes will be analyzed with Exonerate in steps 4, and annotated in step 5 unless the gene models are incomplete due to the presence of mini-exons or other reasons such as genes in pieces or pseudo-genes (to be resolved in step 7). The specific intron Group (I or II) will be assigned with RNAweasel (Beck and Lang, 2009), in step 6.

In step 7, splice sites in gene models derived from Exonerate are refined and mini-exons are identified. As Exonerate allows only a single intron splice-site matrix for splice junctions, combining Group I and Group II intron splice patterns results in an indistinctive, almost neutral matrix. Therefore, MFannot checks and potentially adjusts specific intron boundaries in step 7. Furthermore, Exonerate has difficulties with recognizing and modelling small exons shorter than ~4 codons in length (here referred to as ‘mini-exons’), which occur regularly in mitochondrial genes (for more specific information on Exonerate shortcomings, see ‘Future Developments’, below). As a result, small stretches of otherwise highly conserved amino acid positions can be missing in Exonerate alignments, which is also corrected in step 7 of MFannot (**Figure 2**). Detecting mini-exons is a complex procedure that involves the identification of missing conserved amino acid positions, and scanning of genomic regions that are predicted to contain mini-exons, for best-fitting sequences of the expected size. Technically, this is done by merging each candidate mini-exon with one of the flanking exons and identifying the best candidate from the profile HMM scores for the translated, merged sequences.

In step 8 of protein-gene modelling, MFannot adjusts translation start sites based on matches with profile HMMs, and assesses potential trans-spliced genes (‘genes in pieces’, e.g., (Pereira de Souza et al., 1991; Wissinger et al., 1991; Bonen, 1993; Nadimi et al., 2012)), frameshifts, and in-frame sequence insertions. Start codon identification is based on (i) the range of start positions in the curated multiple protein alignments (used to create the profile HMMs), and (ii) the presence of potential start codons that fall within or close to this range. If ATG codons are absent, known alternative start codons are considered in the order GTG, TTG and ATA, and if no match is found, MFannot leaves a respective comment in the masterfile record.

Step 9 of the procedure is dedicated to finding genes encoding tRNA and other ncRNA genes, such as *rrn5*, *rnpB*, *ssrA*, *rnl* and *rns*, using ERPIN models and CMs (see next section). Finally, step 10 uses profile HMMs to identify less well-conserved proteins among the predicted ORFs.

MFannot stands out from other annotators for listing E-values for intron and gene identification assigned by Infernal and HMM searches. The reporting of E-values is important because it allows the user to make an informed assessment of the results rather than relying on a yes-no answer.

### 2.4 Identification of well-conserved ncRNAs

MtDNA-encoded ncRNAs that are well conserved at the nucleotide level and easy to identify in most species (with the notable exception of Bilateria) are tRNAs and the small and large subunit rRNAs (*rns* and *rnl*, respectively, except for Bilateria and Euglenozoa). MFannot identifies the set of tRNAs with ERPIN models and predicts anticodons and the tRNA identity (amino-acid decoding specificity based on the genetic code that has to be supplied by the user) plus predicted anticodon-codon interactions based on super-wobble rules (for more details see (Lang et al., 2011)). The ERPIN models are sufficiently flexible to recognize unusual structures such as the yeast tRNA(UAG) with an eight-nucleotide anticodon loop that decodes threonine instead of leucine (i.e., in this case, CUN codons have been reassigned from leucine to threonine (Su et al., 2011)). Note, however, that MFannot does not consider identity elements such as the G3:U70 base pair in the tRNA acceptor stem that is recognized by alanyl-tRNA synthetase (Musier-Forsyth et al., 1991; Giegé et al., 1998), which is the molecular basis for mitochondrial codon reassignment in certain yeast species, from Leu to Thr to Ala (Su et al., 2011; Ling et al., 2014). In other words, MFannot will err in rare cases of codon reassignment, both with respect to predicted tRNA identity and protein translation.

**Figure 3:**
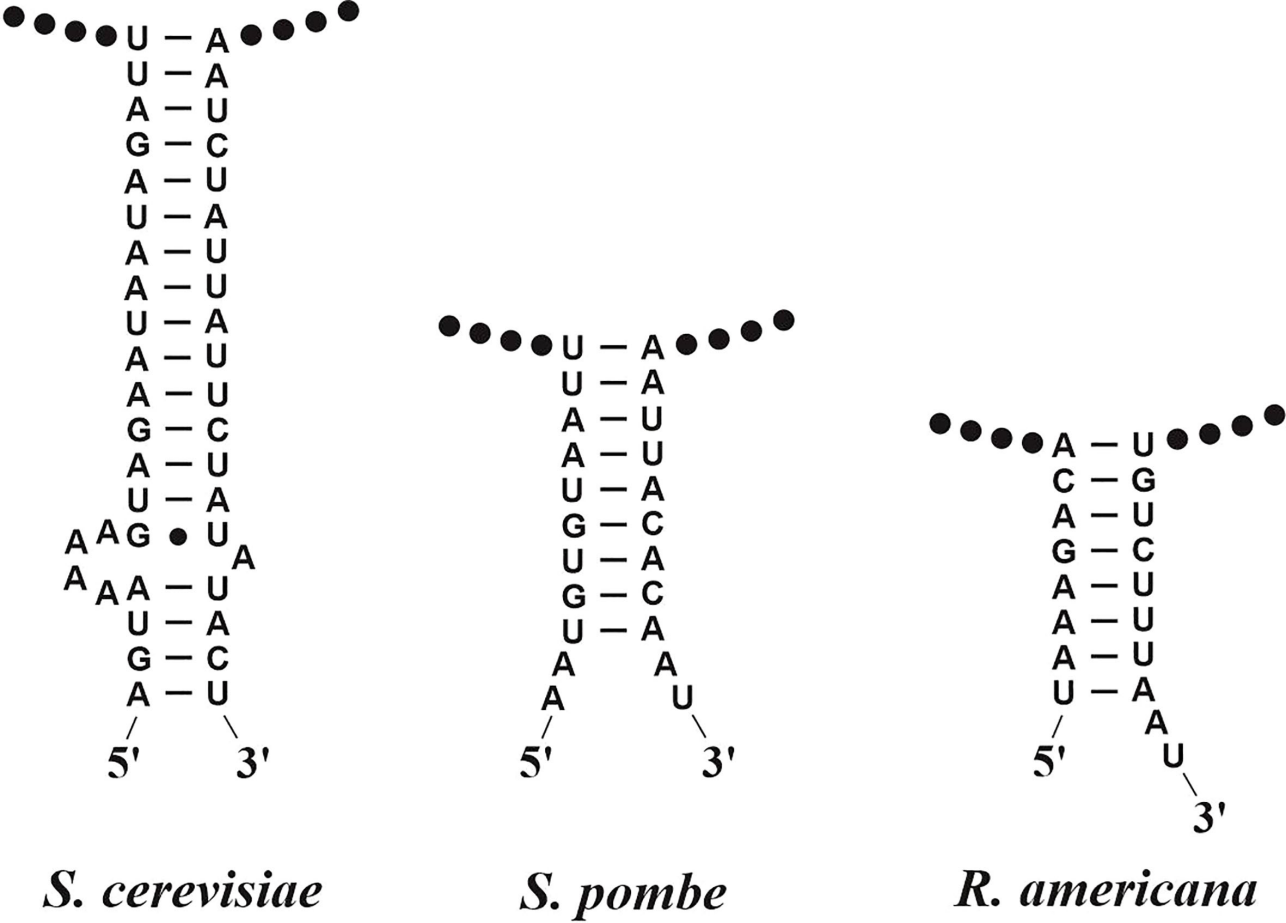
Helical RNA structures joining the 5′ and 3′-terminal regions of mitochondrial large subunit rRNAs. In the three given examples, the RNA termini have been determined experimentally at single-nucleotide resolution (Lang et al., 1987; Lang et al., 1997). The RNA termini are either adjacent to, or within a few nucleotide positions outside the helical region. Note the lack of significantly conserved sequence motifs and the variability of helix shape and length, which renders *in-silico* identification of the termini difficult.

As to rRNAs, the small and large rRNAs are easily identified, even without consideration of 2D interactions. In contrast, the 5S rRNA varies substantially in primary sequence, and therefore requires the use of CMs as described below. In most instances, the small subunit rRNA can be precisely mapped with respect to 5′ and 3′ termini, using an ERPIN model for the corresponding regions. Yet, if introns are present, they are just detected without positioning them in a proper gene model (exon/intron structure). For the latter, manual expert work is required, either by sequence comparison with genes from closely related species (preferentially genes that contain no or few introns) or *via* RNA-seq data that allow positioning exons with reasonable confidence (but see caveats on the use of mitochondrial transcript mapping above). The same applies to identifying *rnl*, yet with one important exception. The 5′ and 3′ termini of the mitochondrial large subunit rRNA carry marginal conservation at the nucleotide level so that the gene’s ends can only be placed in a window of +/- 50 nt. A more precise prediction could be made by pinpointing a terminal helical structure that occurs in almost all instances (**Figure 3**). However, in the absence of significant sequence conservation in this helix, it is virtually impossible to build a eukaryote-wide CM or ERPIN model that uses just this base-pairing information (**Figure 3**). A promising approach to resolving this issue is the construction of clade-specific CMs, as a higher primary sequence conservation can be expected at a shorter evolutionary distance.

### 2.5 Prediction of less-well conserved ncRNAs

**Figure 4:**
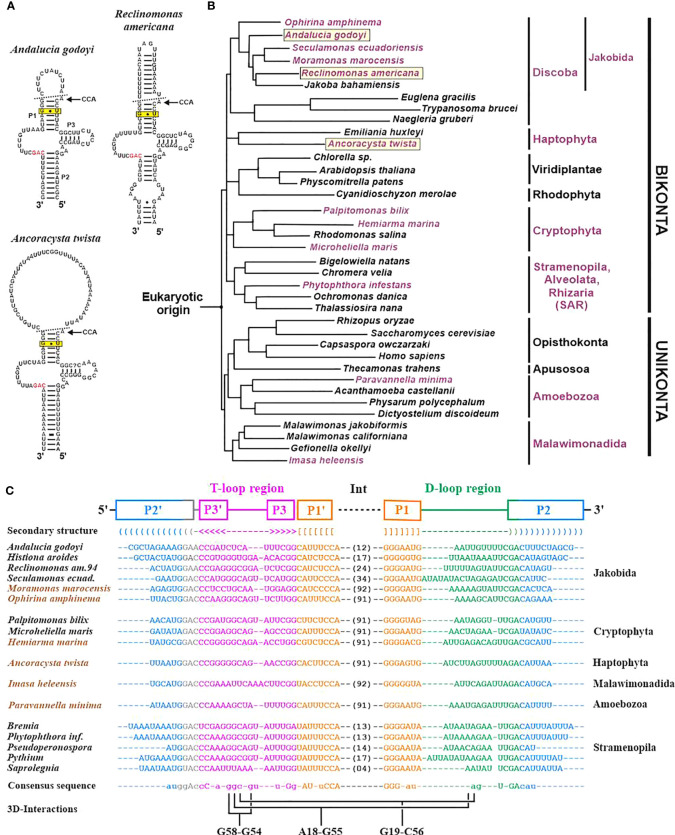
Identification of mitochondrial tmRNA (*ssrA*) genes across eukaryotes. **(A)** Examples of mitochondrial tmRNA 2D structures. The helices P1, P2 and P3 are indicated in the *A. godoyi* 2D structure, in accordance with the sequence alignment in **(C).** The broken line in the 2D structure marks the sites of endonucleolytic processing that give rise to mature tRNA-like 5′ and 3′ ends. The 3′ end undergoes further modification by adding a CCA [51], as indicated in the figure. The invariant G-U pair in helix 3 (boxed) is required to recognize the tmRNA by tRNA synthase alanine. The GAC motif that is invariant across tmRNAs is marked in red. **(B)** Schematic eukaryotic tree (consensus derived from topologies in (Derelle and Lang, 2011; Derelle et al., 2015; Derelle et al., 2016; Janouškovec et al., 2017; Heiss et al., 2021; Tekle et al., 2022; Yazaki et al., 2022)). Names of species containing a mt *ssrA* gene are marked in mauve, and the three species with 2D structures shown in **(A)** are boxed. Mt *ssrA* genes were identified by an exhaustive search of published mitogenomes with a previously published covariance model based on genes from jakobids plus stramenopiles (Hafez et al., 2013a). The search identifies homologs in four eukaryotic lineages formerly thought to lack mt *ssrA* (cryptophytes, haptophytes, malawimonads, and amoebozoans), as well as in numerous additional Oomycota (represented in the tree by a single species, *Phytophthora infestans*). **(C)** Structured alignment of tmRNA sequences. Brown text colour marks the six species for which an *ssrA* annotation in GenBank records is lacking (Shiratori and Ishida, 2016; Strassert et al., 2016; Janouškovec et al., 2017; Yabuki et al., 2018; Bondarenko et al., 2019; Heiss et al., 2021). For further details on previous results and the structural annotation, see (Hafez et al., 2013a). Note that a new version of MFannot (available in June 2023) includes tmRNA-CM searches. During the preparation of the manuscript, we noted a recent publication on two additional jakobid mitogenomes that does not mention the presence of *ssrA* genes (Galindo et al., 2023). As expected due to its almost ubiquitous presence across jakobids (the only current exception is *J. bahamiensis*), *ssrA* genes are present in both *Agogonia voluta* and *Ophirina chinija* mtDNAs (between *rps2* and *trnS*(gcu); E-values of 6.7e-11 and 4.4e-13, respectively).

The three additional ncRNAs that are encoded sporadically in organelle genomes across eukaryotes are 5S rRNA (*rrn5*), tmRNA (*ssrA*), and RNase P RNAs (*rnpB*). Many of these genes remain unidentified in GenBank records, in particular, the genes for tmRNA (**Figure 4**) and RNase P RNA. Still unknown ncRNAs await detection by searching conserved orphan transcripts, followed by comparative phylogenetic modelling with either ERPIN or Infernal.

Among the three mentioned ncRNAs, MFannot identifies 5S rRNA most reliably, based on a CM we developed a few years ago (Valach et al., 2014). The model detects the highly derived structure of *Acanthamoeba castellanii* that was previously identified and characterized biochemically and proposed to represent a 5S rRNA based on expert structure modelling (Bullerwell et al., 2003). Identifying genes of tmRNA is similarly complex as for 5S rRNA but they can be effectively spotted when searching with a published mitochondrion-specific tmRNA covariance model (Hafez et al., 2013a). As the tmRNA CM has so far not been integrated into MFannot, users who did not search separately for this gene could have easily missed it, as documented in **Figure 4C**. This shortcoming will be eliminated in the next version of MFannot. The major remaining challenge is the prediction of RNase P RNA, which according to our preliminary results will require several taxon-specific models (e.g., separate models for yeasts, as well as several other ascomycete and basidiomycete lineages, just to cover the fungal lineage). In fact, it is entirely possible that the reported sporadic presence of less-well conserved and structurally highly variable ncRNAs is in part due to missed identification rather than evolutionary loss. A point in case is fungal mitochondrial RNase P RNA that has an unprecedented structural variability (Seif et al., 2003; Seif et al., 2005).

### 2.6 Current limitations and future developments of MFannot

At the time of writing, two major issues remain to be resolved: mini-exon predictions in protein-coding genes, and modelling of intron-containing genes encoding the small and large subunit rRNAs (*rns* and *rnl*).

**Figure 5:**
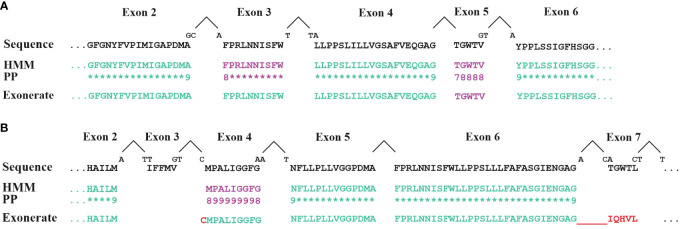
Comparison of exon identification with either HMM or Exonerate. Exons in *cox1* genes (encoding cytochrome c oxidoreductase subunit 1) from **(A)** *Allomyces macrogynus* (GenBank U41288.1) and **(B)** *Morchella crassipes* (GenBank MN542893.2) were identified with either Exonerate (using a single Cox1 protein for the comparison), or HMMER (search with a Cox1 profile HMM in conceptually translated proteins of mitochondrial genes). Lines labelled ‘Sequence’ display the expected, expert-curated protein sequence. Intron positions are marked by a ‘**^**’. ‘PP’ indicates the posterior probability values of HMM searches (‘8’=0.8; ‘9’=0.9; ‘*’=1.0). When codons are split by an intron, the sequence of the split nucleotide is shown above the ‘Sequence’ line. Exons identified with either the default Exonerate settings or with an Hmmsearch at a reporting threshold of <= 1e-5 are shown in green. Additional exons (mauve sequence) were found with low-stringency parameters: Exonerate with the option –proteinwordlen 3 detects the *A. macrogynu*s exon 5 and the *M. crassipes* exon 4. An HMM search using a cut-off e-value between 1e-5 and 0.1 detects the *A. macrogynus* exon 5. Note that *cox1* exons 3 and 7 of *M. crassipes* are missed by both approaches. Exonerate at these low-stringency settings erroneously adds a single cysteine to exon 3 and an incorrect sequence for exon 7 which is in phase with exon 6. The incorrect Exonerate predictions are shown in red.

For initial gene identification and gene structure modelling of **protein-coding genes**, the current implementation of MFannot heavily relies on BLAST and Exonerate. Yet, Exonerate has several shortcomings. Foremost, Exonerate employs pairwise sequence searches for gene-structure prediction rather than a profile built from several sequences that better represents the protein’s diversity. Furthermore, Exonerate uses a dynamic programming algorithm that maximizes a column-wise similarity score (Slater and Birney, 2005), thus applying a generic substitution probability matrix that is unaware of the actual protein evolution (Henikoff and Henikoff, 1992). Consequently, the tool cannot pinpoint highly conserved or invariable amino-acid positions that are missing in a gene prediction. MFannot compensates, to some degree, for Exonerate’s limited search algorithm and evolutionary model by initially identifying proteins from close neighbours with BLAST and handing them over to Exonerate, which improves the predictions. Still, we have noted that Exonerate sometimes does not find mini-exons (**Figure 5A**) and, in other instance, predicts an incorrect exon sequence while passing over the valid one (**Figure 5B**, exons 3 and 7). To resolve this issue, we have developed an MFannot-specific code (**Figure 2**, **step 7**) that identifies potential errors and omissions of mini-exons and makes corrections based on the profile HMM approach. While the current mini-exon detection works well in the majority of cases, for unknown reasons, it sometimes fails when gene structures are complex, calling for an investigation of conditions that cause such failures. Pitfalls include the failure to identify short N-terminal exons that are impossible to resolve with the current approach based on Exonerate and our custom mini-exon identification routine, justifying the development of a more robust algorithm. The presence of unidentified Group I introns exacerbates these issues in certain fungal lineages (e.g., Morchellaceae), which is the reason why we plan to improve our ERPIN intron models in the near future.

The second limitation of the current MFannot version is that ERPIN does not model intron-containing **rRNA genes**. We plan to develop an HMM-based procedure for identifying and modelling rRNA and protein-coding genes. Profile HMMs will be used to scan the genome for conserved regions (as shown in **Figure 5**). Subsequently, exon boundaries will be refined by identifying the best-fitting intron group, either I or II (as described in **Figure 2**, **step 7**). This requires prior intron identification (Prince et al., 2022), which gives hints about the presence of either a mini-exon or, less frequently, twintrons when two introns are seemingly adjacent to each other but not separated by an exon (Hafez et al., 2013b).

To summarize, in future versions of MFannot, the prediction of protein-coding genes will rely exclusively on HMMs, and that of ncRNA genes on HMMs, CMs or ERPIN.

## 3 Methods

### 3.1 Mapping of RNA-seq reads to the mitogenome of *C. posadasii*

To generate the read coverage map shown in **Figure 1**, paired-end Illumina RNA-Seq reads (average length of 170 nt per read) were mapped with HISAT2 (Kim et al., 2015; Kim et al., 2019) to the indexed mitogenome assembly. The resulting uncompressed file was sorted and compressed to BAM format using Samtools (Li et al., 2009). The output from Samtools was then passed to StringTie (Pertea et al., 2015) to assemble into contiguous transcripts.

The corresponding commands were:

1. hisat2-build Coccidioides-posadasii-TGC0611.fna hisat2-index
2. hisat2 –threads 30 –min-intronlen 20 –max-intronlen 30000 -x hisat2-index -1 RNAseq_mito_R1_001.fastq.gz -2 RNAseq_mito_R1_002.fastq.gz | nice -19 samtools sort -m 1G -@ 30 - | samtools view -bh > RNAseq_mito_R1.bam
3. stringtie RNAseq_mito_R1.bam -p 20 –rf -o test.gtf -f 0.1 -m 90 -j 3 -c 3 –conservative

The resulting files were loaded into IGV for visualization (Thorvaldsdóttir et al., 2013).

### 3.2 Comparison of profile HMM and Exonerate for *cox1* gene modelling

*Cox1* exon sequences of the *Morchella crassipes* M10 and *Allomyces macrogynus* mitogenomes were identified with either Exonerate or profile HMM searches. For the profile HMM, the COX1 protein model currently used by MFannot was searched against the six conceptually translated reading frames using HMMER (v3.3.2) with and without the heuristic filters. For Exonerate, a procedure similar to that of MFannot was used. First, the best candidate protein was identified from the MFannot collection using BLAST+ (v2.13.0). This protein sequence was then searched against the mitogenome using Exonerate (v2.2.0), using parameters and splice models used in MFannot. For *M. crassipes*, the maximum intron length was set to 20,000 nucleotides to find the complete gene in a single hit.

## Data availability statement

The original contributions presented in the study are included in the article/supplementary material. Further inquiries can be directed to the corresponding author.

## Author contributions

BL, NB, PR, and GB developed the conceptual framework of MFannot, and NB wrote and then updated the Perl code over a 16 years period, and created a containerized version of MFannot and deployed it in CBRAIN (Sherif et al., 2014). MS provided informatics and coding support, GB tested the various MFannot versions and provided suggestions for improving the tool, and SP conducted the comparison between Exonerate and profile HMM exon predictions. All authors contributed to the article and approved the submitted version.

## Availability of informatics tools

The code for MFannot, RNAweasel and Flip is available *via* separate GitHub repositories (Brossard et al., 1996; Beck and Lang, 2009; Beck and Lang, 2010). MFannot is also available within CBRAIN (Sherif et al., 2014), an open-source platform that allows using MFannot with various extended options, such as modification of E-value cutoffs. Note that the execution of all tools mentioned in this paper requires a Linux system, and knowledge of command-line program execution. The authors encourage people who wish to participate in the further development of MFannot to contact BFL *via* email.
